# Poor Hand Hygiene Procedure Compliance among Polish Medical Students and Physicians—The Result of an Ineffective Education Basis or the Impact of Organizational Culture?

**DOI:** 10.3390/ijerph14091026

**Published:** 2017-09-07

**Authors:** Marta Wałaszek, Małgorzata Kołpa, Zdzisław Wolak, Anna Różańska, Jadwiga Wójkowska-Mach

**Affiliations:** 1 State Higher Vocational School in Tarnów, 33-100 Tarnów, Poland; mz.walaszek@gmail.com (M.W.); malgorzatakolpa@interia.pl (M.K.); zdzich_w@interia.pl (Z.W.); 2 Chair of Microbiology, Faculty of Medicine, Jagiellonian University Medical College, ul. Czysta 18, 31-121 Kraków, Poland; rozanska@ifb.pl

**Keywords:** hand hygiene, healthcare-associated infections, medical students, physicians

## Abstract

*Objective*: The objective of the study was to examine the knowledge of Polish physicians and medical students about the role of hand hygiene (HH) in healthcare-associated infection (HAI) prevention. *Study design*: A survey was conducted using an author-prepared questionnaire, which was filled out on the first day of hospital work (or internship) by newly admitted physicians who had worked in other hospitals and students of different medical schools in Poland. *Methods*: 100 respondents participated in the study: 28 students, 18 medical interns and 54 physicians. *Results*: As many as 3/4 of physicians and students did not use the HH techniques correctly. The respondents declared that they perform HH in the following situations: 74.4% of respondents before an aseptic task; 60.8% before patient contact; 57.0% after patient contact; 11.5% after body fluid exposure risk, and only two respondents (1.1%) after contact with patient surroundings. 64% of respondents declared that their supervisor checked their knowledge of the HH technique when they were touching patients, but their supervisors checked the five instances for HH only in the case of 27 respondents (27%). Students experienced any control of HH in the workplace less often. Interns and physicians mentioned that the most important preventive action in HAI is HH, but for students it is the use of gloves. *Conclusions*: The level of knowledge and skills of physicians and students in the field of HH is insufficient. Deficiencies in skills and knowledge of HH were identified as early as at the level of the first internship.

## 1. Introduction

The first recommendations for hand hygiene (HH) in medical settings were initially based on the recommendation to wash hands with soap and water and rinsing hands with an antiseptic agent was recommended only if water was unavailable. However, the study by Rotter [1] on hand disinfection efficiency revolutionized the approach to HH and had been recommended for hand disinfection for many years in place of washing hands, also in Poland [2]. HH should be applied at five moments as indicated by the WHO: (1) before patient contact; (2) before an aseptic task; (3) after body fluid exposure risk; (4) after patient contact; (5) after contact with patient surroundings [2,3]. It was confirmed by Allagranzi et al. [4] that the WHO campaign in the field of HH is effective in numerous studies that were subjected to meta-analysis.

However, medical personnel face many difficulties in the implementation of HH rules. These difficulties have been described in various studies from around the world. HH is not always conducted in accordance with the principle of the 5 Moments for HH, and compliance with these rules is estimated at around 40% of needs [5]. Particularly low compliance rates were obtained in studies conducted among interns [6,7]. The results of studies conducted by different Polish authors also show low compliance rates with HH recommendations [8,9,10,11,12]. It is difficult to clearly state what causes the problems as regards compliance with HH rules in Poland. Some authors point to the flawed organization of the infection control system, which can result from the short post-transformation history [13], and others indicate the national culture in the hospital-acquired infection (HAI) surveillance system, which is specific to a given country and the planning and operation of which may have a positive impact on HAI prevention practices, including HH [14,15,16]. In the literature it is more often noticed that the success of HH campaigns is affected by the contextual factors of the organization, such as the involvement of the hospital administration and the good example presented by managers and senior managers [17,18]. However, in countries where local cultural factors are ignored such campaigns may not produce the expected results [19,20,21,22,23].

The main objective of this 2016 study was to examine the knowledge and skills of medical students and physicians in the field of HH in the context of HAI prevention, using a standardized questionnaire survey, and to additionally identify the possible cause of the current condition via a qualitative interview survey. Moreover, an observational study of HH practices was performed in order to check the data received from the questionnaire and the interviews.

## 2. Methods

Three research methods were employed in the study: a quantitative method, i.e., the use of a questionnaire designed by the authors, a qualitative method involving interviews (based on the structured questionnaire), and observation of the Ayliffe handwashing technique. The questionnaire surveys and interviews were anonymous. The questions used in the questionnaire survey and in structured interviews are presented in Table 1. All groups (students, interns and physicians) took part in the questionnaire survey—100 respondents, comprising 65 women (56.9%) and 35 men (48.6%). In this group, there were 28 medical students (28%), 18 intern physicians (18%) and 54 physicians (54%). The structured interviews were conducted only with students and interns since physicians refused to participate in this part of the study. Interviews were recorded and transcripts were later prepared by the interviewer. In addition, between 2010 and 2017, the Infection Prevention and Control Team reviewed the Ayliffe HH technique among physicians and students hired.

The study was conducted in 2016 among physicians and medical students undertaking internships in a 500-bed specialist multiprofile hospital located in Tarnow (south east Poland). Three different groups of respondents took part in this study: medical students, intern physicians, and newly hired physicians at the hospital.

Medical students participating in the study were students who had completed the first year of studies and were selected for a holiday medical internship in the hospital where the study was conducted. Intern physicians (the second group) are physicians who graduated from a 5-year medical school and passed the National Medical Exam and work in a hospital of their choice for 1 year before their independent medical practice. The third group consisted of physicians who were newly admitted to the hospital in which this study was conducted (in this group there were both residents, i.e., physicians during specialization and fully qualified ones).

In the case of students and intern physicians, the assumption was made that they had studied at or graduated from medical schools located in various Polish cities. Both the students and physicians chose the study hospital for their internship because it was close to the place of their permanent residence. This allowed the authors to obtain a diverse group of people who had studied at different medical schools in Poland.

In the questionnaire, responses to the questions concerning HAI prevention were rated by assigning the rank to individual activities from 1 (invalid) to 7 (very important) according to the Likert scale. As for the question concerning difficulties in compliance with the HH procedure, points were assigned for each action according to the Likert scale ranging from 1 (no obstacle) to 5 (a very big obstacle).

The group of 127 physicians was covered by the observational study (no medical students were included). Among them there were 72 physicians, including the group of 54 taking part in the questionnaire survey, and 55 intern physicians, including the group of 18 taking part in the questionnaire. The hospital, in which this study was conducted, refers every newly-hired employee to the team in order to verify their ability to perform the Ayliffe HH technique.

For statistical analysis, all factors that may influence the proper use of HH by physicians, such as sex, age, education, seniority, and place of work were considered as independent variables. Statistical analysis of the collected material was carried out with the use of the IBM Statistical Package for the Social Sciences (IBM SPSS v.4, Armonk, NY, USA) and Microsoft Office Excel (2003, Redmond, Washington, DC, USA). The description of data on the whole population surveyed was compiled with the use of basic statistical parameters, such as mean and standard deviations, confidence intervals, minimum and maximum. In order to compare the incidence of qualitative variants in several populations, the chi-square test of independence and measurement error or uncertainty indicators were used.

## 3. Results

### 3.1. Structured Questionnaire

Activities related to HH were pointed out by the respondents as the most important in the area of HAI prevention—in this case the highest estimated mean value was 5.9 (median, Me 7). Respondents equally highly evaluated the use of disposable gloves (mean 5.4, Me 6) and adherence to aseptic technique (mean 5.3, Me 6). Substantial differences in the perception of HAI prevention were observed depending on the occupational group. The most important activity indicated by interns (I) and physicians (P) was HH (on average 6.2 and 6.0), and according to medical students (MS) it was the use of gloves (average 6.6). Detailed results are presented in Table 2.

However, the knowledge of HH demonstrated by the respondents in their answers to the open question appeared to be unsatisfactory. Only 79 respondents answered this question. From the situations (moments) in which (according to the WHO) HH should be performed, only 59 respondents (74.7%) indicated the moments before clean/aseptic tasks; 48 (60.8%) before patient contact; 45 (57.0%) after patient contact; 20 (25.3%) after body fluid exposure risk, and only 2 (2.5%) after contact with patient surroundings (Table 3). The interns gave most correct answers: 80% pointed to three situations in which HH was required: before patient contact; before clean/aseptic tasks; and after patient contact. The group of physicians most frequently mentioned HH before clean/aseptic tasks (85.7%) (Table 3).

As regards the question, “Has your Ayliffe technique ever been checked?”, only 64 respondents (64%) confirmed that such an inspection ever took place. An even worse result was obtained when the question about the 5 Moments for HH was asked: “Has your ability to recognize the 5 Moments for HH ever been checked?” Only 27 respondents (28%) answered affirmatively.

In order to further investigate the abovementioned issue, the following question was asked: “Who checked your Ayliffe hand washing technique?” We received answers from only 64 respondents. The next question was as follows: “Who reviewed the situations in which you apply the 5 Moments for HH?” Only 26 respondents answered. In most cases the control of HH was conducted by the nurse in charge. The nurses in charge conducted the control of HH in 25 cases (39.1%) out of the 64 respondents. Similarly, the nurses in charge controlled the 5 Moments for HH in 12 cases (46.2%) of respondents out of 28 respondents who reported that they experienced any such control. The study shows the low participation of teachers (internship supervisors) in carrying out such inspections. In addition, the Head Physicians (department managers) showed little action in the field of HH control. The most active group comprised nurses in charge, especially in relation to the group of students whom they supervised during internship after the first year of medical studies (Table 4). The interviews highlighted the existence of barriers between different medical personnel groups concerning the necessity to remind them about performing HH in the required situations.

Respondents were asked the following question: What situations encountered in everyday work influence compliance with HH recommendations? In most cases, respondents pointed to difficulties in accessing soap, towels and disinfectants, and the inadequate quality of disinfectants, which caused irritation. Respondents also showed a skeptical approach to HH, forgetting about HH, and the belief that the use of gloves is sufficient (Table 5).

Respondents were asked an open question: What would encourage medical personnel to obey HH rules better? The highest proportion of answers pointed to the need for a greater number of courses (35 respondents, 24.8%). Other answers indicated access to good quality disinfectants (28 respondents, 19.9%), a better supply of HH products (21 respondents, 14.5%), and others listed in Table 6.

### 3.2. Structured Interviews

None of the students could list the “5 moments for HH”, while among the interns this percentage was lower and amounted to 82%. Medical students favored hand washing and use of gloves (83%) as essential HH practices. Interns leaned towards hand disinfection (47%), hand washing (24%) or washing and disinfection (29%). Both medical students and interns stated that patients had never asked them to apply HH (56%); however, if it had happened, they would have been embarrassed. 9% of respondents would have felt obliged to comply and almost half would have been surprised or embarrassed.

Only 29% of medical students confirmed in their interviews that the educational program of their studies covered elements involving hand hygiene. As for interns, this proportion was higher and amounted to 65%. Medical students most often mentioned theoretical hand hygiene classes (21%). In the course of their education, theory was rarely combined with practice (7%). The interns more often pointed out theory (41%), practice (35%) and combining theory with practice (23%).

As regards hand hygiene practices, the interns complained most frequently about low availability of hand hygiene preparations (41%), requested more training (35%) and substances of better quality (24%). Medical students needed HH trainings the most (61%).The interns often observed situations when medical staff did not comply with HH guidelines (77%). Medical students witnessed such events much less frequently (29%).Neither interns (94%) nor medical students (61%) were inclined to remind others about the necessity to conduct hand hygiene in the required situations.

### 3.3. Observational Study

In addition to the results of the diagnostic survey described above, the inspection of the Ayliffe HH technique was conducted in the hospital from 2010 to 2017 among 127 physicians, including a group of 54 newly-hired physicians taking part in the questionnaire survey. The review was conducted on the day a physician was collecting the documents required to start work. One of the required documents is the so-called "new employee orientation checklist". It is required that the infection control (IC) team confirms on this checklist that an applicant has mastered skills in the field of HH. In 2010–2017, 127 physicians were checked on the correct use of the Ayliffe HH technique. An unfavorable situation in this area was recorded and it remained unchanged for many years as the distribution in the examined group over the years has not shown significant statistical differences between the HH skills in individual years (*p* = 0.327). The number of people who correctly performed HH are as follows: eight interns out of 55 (14.5%) and 24 out of 72 physicians (33.3%). The differences between the professional groups of physicians were statistically significant (*p* < 0.05) (Table 7).

## 4. Discussion

The use of triangulation, involving three methods to collect data (survey, interview, observation), was applied in this study to deepen research in the field of HH and, in particular, to broaden the knowledge of the context of the phenomenon studied. Both quantitative (survey, observation) and qualitative (interviews) studies responded to the questions asked by the authors of this paper regarding poor compliance with HH rules by physicians. Although the long history of HAI surveillance in the world indicates HH as a primary activity in HAI prevention, many scientific reports indicate deficiencies in knowledge in this area [2,6,7,18,24]. Also, on the basis of studies conducted in Poland, it can be concluded that compliance with HH rules by medical personnel is far from what should be expected [8,9,10,11,12]. The lack of basic knowledge about HH is already apparent in the case of first year students who should have basic knowledge in the field of HH before their first patient contact. In this study, students incorrectly indicated the importance of using protective gloves as the most important action in HAI prevention. These results may indicate that the HH rules are not promoted strongly enough in medical schools. It is hard to say whether students’ opinions were guided by patient welfare, or whether they were worried about their own health before patient contact; hence, the lack of experience or knowledge in this area means that they chose the simplest solution. Similar results were obtained in another study on the effectiveness of the implementation of HH programmes [25].

In the study conducted, only a few supervisors controlled the technical skills and frequency of HH of their subordinate personnel. This situation may mean that a low rank (value) is assigned to HH as the basic skill required in patient contact. It is true that numerous studies demonstrated the effectiveness of audits in this area [18,25,26,27,28].

In the study carried out, it was important for the authors to receive an answer to the following question: Who inspected the Ayliffe technique and the 5 Moments for HH? The objective behind this question was to gather information from respondents on the extent to which their knowledge and skills in the field of HH were strengthened by their supervisors carrying out checks. In most cases, this control was conducted by nurses in charge, and the inspection was less often performed by supervisors and Head Physicians.

Interns emphasized the fact that they acquired HH skills gradually during internship in different hospitals. They also emphasized the role of nurses in acquiring practical skills in the field of hand hygiene. Numerous studies underline the importance of organizational structure in the effective implementation of HH programmes [17]. Positive leadership at the departmental level appears to be a prerequisite for medical personnel’s compliance with HH rules. Supervisors and senior physicians should be aware of their role and demonstrate HH skills as role models for novice medical professionals [20,29]. In this study, interviews with physicians and students demonstrated the existence of barriers between professional medical groups as regards the need to remind them to perform HH in the required situations. In medical students’ statements, one can notice a reference to the extensive experience of medical personnel and respect for their knowledge, but also socialization taking place in medical schools, which prevents questioning even negative behaviors. Students tend not to speak up in situations when it is necessary to point out that someone is not following HH rules. Undoubtedly, this situation is related to the national culture and the consequent trend towards building hierarchical structures based on a large power distance [15,19,30].

This study revealed the great ignorance of physicians and students with regard to the 5 Moments for HH. During interviews some physicians complained about the lack of HH procedures at their workplace and students talked about negative situations which they had observed during their internship involving senior physicians and supervisors and the lack of HH classes in the course of their studies. Numerous studies show that the level of HH among students and physicians is unsatisfactory. It also concerns other countries [6,7,8,9,14].

The physicians and students who took part in the study indicated difficulties in conducting HH, but it should be strongly emphasized that the results of these tests do not refer specifically to the hospital where the study was conducted, but rather to the general problems in different Polish hospitals, where the studied group of physicians and students worked. This situation is very disturbing and may suggest that hospital managers implement austerity policies in the field of HH. Among the activities which, according to respondents, would encourage medical personnel to comply with the HH rules were better education, better access to high quality disinfectants, and better supply of HH products. These results are in part consistent with those of Borg et al. [31] De Bono et al. [15] focus on HH practices that are the central element of the culture. Culture in this view is defined as the “collective programming of mind”, and is characterized by high stability [19]. This means that an attempt to change the practices may be treated by individuals or groups as a threat that may question the foundations of HH in order to maintain actions and status within the structure of authority. It should be emphasized that the strong resistance to change in Poland is caused by the high level of risk experienced by medical personnel when facing new, unknown and uncertain situations [19]. Resistance to change among Head Physicians and senior managers is the result of uncertainty as to the relevance of new practices which delays and increases the effort to implement evidence-based changes [15].

## 5. Conclusions

(1) The level of knowledge and skills of physicians and medical students in the field of HH is insufficient and needs to be supplemented with pre/diploma training, and should also be part of postgraduate training.
(2) The participation of managers of hospital departments and teachers in supervising HH of new physicians and students is inadequate, which may be one of the reasons behind the poor situation in this field.
(3) Physicians and students report that Polish educational programmes for higher medical schools do not contain sufficient training in hand hygiene.
(4) Further studies on the cultural determinants of compliance with HH rules in the medical field are needed to enable their effective implementation.

## Figures and Tables

**Table 1:** Questions used in the questionnaire survey and in structured interviews.

**Questionnaire Survey—Questions about Respondents’ Knowledge of Selected Aspects of HH**
what are the main measures aimed at preventing HAIs?
when should they be performed? (‘5 Moments for HH’)
whether anyone (and if yes–who) in the hospital inspected their Ayliffe HH technique
whether anyone (and if yes–who) in the hospital inspected the 5 Moments for Hand Hygiene
what situations encountered in everyday work influence compliance with HH recommendations?
what would encourage medical personnel to obey HH rules better?
**Structured Interviews—Questions**
can you list the ‘5 Moments for HH’?
how do you practice HH while working with patients?
how would you feel if a patient made a remark about omitting hand hygiene?
what is the level of compliance with HH recommendations among healthcare workers?
were HH recommendations included in your studies?
was it only a theoretical or also a practical approach?
what could help us build a positive attitude towards HH?
have you experienced any severe situations concerning disobeying the HH rules?
would you report anybody who disobeys the HH rules?

**Table 2:** Main measures to prevent healthcare associated infections according to professional groups.

Activities Related to HAI Prevention	Professional Groups	*N*	Average Values in Groups	Median	Standard Deviation	95% CI
Hand-hygiene	Medical student	28	5.6	6.0	2.71	4.649, 6.494
	Medical intern	18	6.2	7.0	2.71	4.873, 7.571
	Physician	54	6.0	7.0	2.78	5.241, 6.759
Use of disposable gloves	Medical student	28	6.6	7.0	1.52	6.016, 7.198
	Medical intern	18	5.0	5.5	2.57	3.724, 6.276
	Physician	54	4.9	5.0	2.41	4.229, 5.548
Adherence to aseptic technique	Medical student	28	5.2	5.0	1.97	4.451, 5.978
	Medical intern	18	5.5	6.0	2.50	4.255, 6.745
	Physician	54	5.3	6.0	2.63	4.616, 6.051

**Table 3:** Answers to the multiple choice question: List situations in which a healthcare worker should perform hand hygiene according to the 5 Moments for Hand Hygiene with the division into professional groups. Number of respondents: 79, no answer: 21.

5 Moments for Hand Hygiene		Medical Student	Medical Intern	Physician	Total
Before patient contact	In a professional group *N* (%)	13 (59.1)	12 (80.0)	23 (54.8)	48
	% of total	16.5%	15.2%	29.1%	60.8%
Before clean/aseptic tasks	In a professional group *N* (%)	11 (50.0)	12 (80.0)	36 (85.7)	59
	% of total	13.9%	15.2%	45.6%	74.7%
After body fluid exposure risk	In a professional group *N* (%)	9 (40.9)	2 (13.3)	9 (21.4)	20
	% of total	11.4%	2.5%	11.4%	25.3%
After patient contact	In a professional group *N* (%)	13 (59.1)	12 (80.0)	20 (47.6)	45
	% of total	16.5%	15.2%	25.3%	57.0%
After contact with patient surroundings	In a professional group *N* (%)	0 (0.0)	1 (6.7)	1 (2.4)	2
	% of total	0.0%	1.3%	1.3%	2.5%
Total	In a professional group *N* (%)	22 (27.8)	15 (19.0)	42 (53.2)	79 (100.0)
	% of total	27.8%	19.0%	53.2%	100.0%

**Table 4:** Answer the question: Who checked your Ayliffe hand washing technique and the 5 Moments for Hand Hygiene [2,3]?

Professional Groups	Who Reviewed the Ayliffe Hand Washing Technique?				Who Checked the ‘5 Moments for Hand Hygiene’?			
	*N* (%)				*N* (%)			
	Head Physician	Nurse in Charge	Teacher/Internship Supervisor	Total	Head Physician	Nurse in Charge	Teacher/Internship Supervisor	Total
Medical student	2 (16.7)	9 (75.0)	1 (8.3)	12 (100)	0 (0.0)	5 (100.0)	0 (0.0)	5 (100)
Medical intern	0 (0.0)	3 (30.0)	7 (70.0)	10 (100)	0 (0.0)	2 (50.0)	2 (50.0)	4 (100)
Physician	17 (40.5)	13 (32.0)	12 (28.6)	42 (100)	6 (35.35)	5 (29.4)	6 (35.3)	17 (100)
Total	19 (29.7)	25 (39.1)	20 (31.1)	64 (100)	6 (21.1)	12 (46.2)	8 (30.85)	26 (100)
	Lack of answer *n* = 36				Lack of answer *n* = 74			
	Chi-square of Pearson *p* < 0.01				Chi-square of Pearson *p* < 0.05			

**Table 5:** Answers to the question: What situations encountered in everyday work affect compliance with HH recommendations? SD—standard deviation; 95% CI.

Situation or Opinion That Impedes Hand Hygiene	Number	Mean	SD	95% CI
Lack of soap and towels	54	4.5	0.79	4.265, 4.698
Insufficient amount of disinfectants	54	4.3	0.69	4.125, 4.505
Disinfectants causing irritation	54	3.7	0.82	3.461, 3.909
Skeptical attitude to hand hygiene	54	3.6	1.27	3.247, 3.938
Forgetting about hand hygiene	54	3.6	1.09	3.276, 3.872
The use of gloves is sufficient	54	3.5	1.11	3.803, 3.197
Disagreement with hand hygiene recommendations	54	3.3	1.28	2.928, 3.627
Overload of activities	54	3.2	0.95	2.964, 3.480
Lack of specific hand hygiene rules	54	3.2	1.12	2.897, 3.510
Lack of pattern of conduct among colleagues and supervisors	54	3.1	1.12	2.749, 3.362
Lack of a reward and punishment system	54	2.2	1.01	1.926, 2.481

**Table 6:** Answers to the multiple choice question: What would encourage medical personnel to obey HH rules better? Number of respondents: 79, no answer: 21.

Suggestions for Improving Hand Hygiene	Answers	
	*N* (%)	Percent of Observations
More training	35 (24.8)	42.2%
Hand hygiene products of good quality	28 (19.9)	33.7%
Better supply of hand hygiene products	21 (14.9)	25.3%
A system of rewards and punishments	15 (10.6)	18.1%
More frequent inspections	14 (9.9)	16.9%
Good models	10 (7.1)	12.0%
Increasing the number of personnel	9 (6.4)	10.8%
Personnel’s positive attitude	5 (3.5)	6.0%
Verbal reprimands	4 (2.8)	4.8%
Total	141 (100.0)	169.9%

**Table 7:** Summary of observations on compliance with the Ayliffe hand washing technique made between 2010 and 2017 with division into professional groups.

Year	Non-Compliant *N* (%)	Compliant *N* (%)	Total *N* (%)	*p* *
2010	8 (8.4)	4 (12.5)	12 (9.4)	0.327
2011	11 (11.6)	3 (9.4)	14 (11.0)	
2012	3 (3.2)	1 (3.1)	4 (3.1)	
2013	13 (13.7)	4 (12.5)	17 (13.4)	
2014	15 (15.8)	6 (18.8)	21 (16.5)	
2015	29 (30.5)	4 (12.5)	33 (26.0)	
2016	11 (11.6)	9 (28.1)	20 (15.7)	
2017	5 (5.3)	1 (3.1)	6 (4.7)	
Total	95 (100.0)	32 (100.0)	127 (100.0)	
**Professional Group vs. Ayliffe Hand Washing Technique**				
Physician	48 (66.7)	24 (33.3)	72 (100.0)	<0.05
Intern	47 (85.5)	8 (14.5)	55 (100.0)	

* From Chi-suqare of Pearson test.
